# Comparisons of normal saline and lactated Ringer’s resuscitation on hemodynamics, metabolic responses, and coagulation in pigs after severe hemorrhagic shock

**DOI:** 10.1186/1757-7241-21-86

**Published:** 2013-12-11

**Authors:** Wenjun Z Martini, Douglas S Cortez, Michael A Dubick

**Affiliations:** 1 US Army Institute of Surgical Research, JBSA Ft, 3698 Chambers Pass, Sam Houston, TX 78234, USA

**Keywords:** Hemorrhagic shock, Oxygen metabolism, Coagulation, Pre-hospital resuscitation and Thromboelastrograph®

## Abstract

**Background:**

Ongoing improvements in trauma care now recommend earlier use of blood products as part of damage control resuscitation, but generally these products are not available at far forward battlefield locations. For the military, questions continue to arise regarding efficacy of normal saline (NS) vs. lactated Ringer’s (LR). Thus, this study compared the effects of LR and NS after severe hemorrhage in pigs.

**Methods:**

20 anesthetized pigs were randomized into control (n = 6), LR (n = 7), and NS (n = 7) groups. Hemorrhage of 60% estimated total blood volume was induced in LR and NS groups by removing blood from the left femoral artery using a computer-controlled pump. Afterwards, the pigs were resuscitated with either LR at 3 times the bled volume or the volume of NS to reach the same mean arterial pressure (MAP) as in LR group. Hemodynamics were measured hourly and blood samples were taken at baseline (BL), 15 min, 3 h and 6 h after resuscitation to measure changes in coagulation using thrombelastograph®.

**Results:**

MAP was decreased by hemorrhage but returned to BL within 1 h after resuscitation with LR (119 ± 7 ml/kg) or NS (183 ± 9 ml/kg, p < 0.05). Base excess (BE) was decreased by hemorrhage; resuscitation with LR recovered BE but not with NS. Total peripheral resistance was decreased with NS and LR, with a larger drop shown in NS. Serum potassium was increased with NS, but not affected with LR. Coagulation changes were similar between LR and NS.

**Conclusions:**

NS may be inferior to LR in resuscitation due to its vasodilator effects and the risks of metabolic acidosis and hyperkalemia.

## Background

Normal saline (NS) and lactated Ringer’s (LR) solution have been used as crystalloid fluids for decades [1,2], but controversies continue as to which crystalloid is best. Although damage control resuscitation was introduced recently to initiate early use of blood products for severely injured hypotensive trauma patients [3-6], blood products are not generally available at pre-hospital and far forward military settings. Crystalloids remain to be used as resuscitation fluids under these conditions with survival benefit [7]. NS contains 154 mM Na^+^ and Cl^-^, with an average pH of 5.0 and osmolarity of 308 mOsm/L. LR solution has an average pH of 6.5, is hypo-osmolar (272 mOsm/L), and has similar electrolytes (130 mM Na^+^, 109 mM Cl^-^, 28 mM lactate, etc.) to plasma; thus, it was considered a more physiologically compatible fluid than NS. Since its initial use in 1831 for treating cholera [8,9], NS has been recommended as therapeutic intravenous (IV) fluid in various clinical situations, such as kidney transplantation, blood storage, and blood transfusion [10,11], although development of hyperchloremic acidosis is commonly observed [12-14]. LR’s acid base balance is superior to that of NS’s [15,16], but it has not been approved by the Association of American Blood Bank for use with blood products in the same iv-line [11]. Thus, it is necessary and clinically relevant to comprehensively and systemically examine the resuscitative effects of these two crystalloids.

Limited experimental evidence has contributed to the difficulty over the selection of resuscitation fluids. LR’s better acid base balance and survival has been shown in a rat model with massive hemorrhage [17] and an unanesthetized swine model with rapid hemorrhage [18], suggesting the superiority of LR under severe trauma setting. In pigs with uncontrolled hemorrhage, Kiraly et al. showed that LR resuscitation was associated with hypercoagulability and less blood loss over a 2-hour post-injury period [19]. It is unclear whether the hypercoagulability is transient or is likely to be a concern. On the other hand, a recent multicenter survey of prehospital intervention at a combat zone showed that IV fluids administered in the field favored the use of NS by 73% to 17% LR [20], even though Hextend® (manufactured by BioTime, California, USA, with 6% hetastarch, a mean molecular weight of 670 kD and a degree of hydroxyethyl group substitution of 0.75 (HES 670/0.75)) is the fluid recommended by the Committee on Tactical Combat Casualty Care [21]. In addition, the Israeli Defense Force has recently recommended LR as initial resuscitation of their combat casualties [22]. Thus, the greater use of NS by medics than the recommendation for Hextend® [20,21], recommendation for pre-hospital LR by the Israeli Defense Force [22], and the inquiry from the US Army’s Combat Developer’s office regarding NS vs. LR for pre-hospital resuscitation, all together prompted this current study. In order to assess more comprehensively the physiological and biochemical effects of LR and NS, we compared the resuscitative effects of LR and NS on hemodynamics, oxygen metabolism, and coagulation in a swine model with severe hemorrhagic shock over a 6 h post resuscitation period; the time period in which most trauma patients die from hemorrhage [23].

## Methods

### Experimental design

This animal study was approved by the Institutional Animal Care and Use Committee of the US Army Institute of Surgical Research and was conducted in compliance with the Animal Welfare Act and the implementing Animal Welfare Regulations and in accordance with the principles of the Guide for the Care and Use of Laboratory Animals. A total of 20 pigs (34.6 ± 1.2 kg) were randomly allocated into three experimental groups: sham control (n = 6), hemorrhage with LR resuscitation group (LR, n = 7), and hemorrhage with NS resuscitation group (NS, n = 7). After an overnight fast, the animals were pre-anesthetized with Glycopyrrolate (Glycopyrronium bromide, 0.1 mg/kg, America Regent, Shirley, NY, USA) and Telazol® (Tiletamine HCl and Zolazepam HCl, 6 mg/kg, Pfizer, NY, NY, USA). Anesthesia was induced via a facemask with approximately 5% Isoflurane (Forane, Baxer, Deerfield, IL, USA) in 100% Oxygen. The pigs were then intubated with a cuffed endotracheal tube (7.5 mm, Rusch, Teleflex Medical, Research Triangle Park, NC). During surgical instrumentation, anesthesia was maintained with 1% to 3% isoflurane in 30% oxygen in air using a ventilator and monitor (Fabius gas anesthesia system and Infinity Explorer monitoring system, Draeger Medical, Telford, PA). Tidal volume was set at 7 ml/kg with a rate of 25 breaths/minute. Ventilation was adjusted to reach an end tidal pCO_2_ of approximately 40 mmHg at baseline. Afterwards, no adjustments in ventilation setting were made throughout the experiment. Polyvinyl chloride catheters were inserted into the thoracic aorta via the carotid artery to measure mean arterial pressure (MAP), systolic and diastolic blood pressure, and heart rate. A Swan-Ganz thermodilution catheter was inserted in the pulmonary artery via the left jugular vein to measure cardiac output and temperature. The right femoral artery was cannulated for arterial blood sampling and induction of bleeding. The right jugular vein was cannulated for venous blood sampling. The left femoral vein was cannulated for resuscitation. The right femoral vein was cannulated for IV anesthesia during the study. No splenectomy was performed in this study.

**Figure 1:**
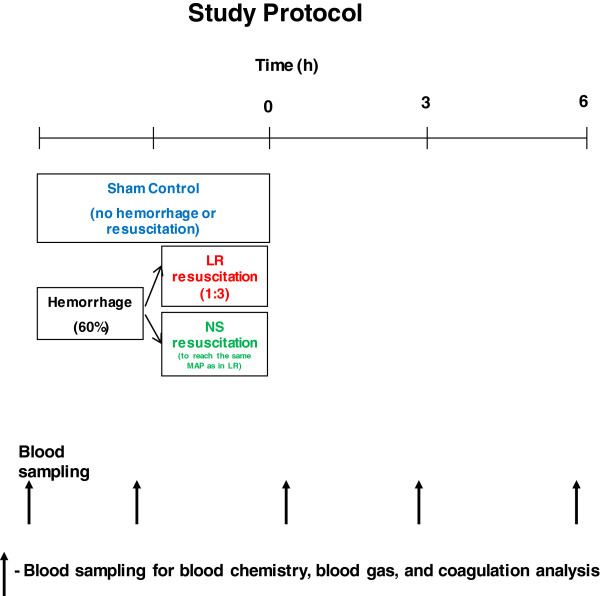
Experimental protocol.

Upon completion of surgical procedures, anesthesia was switched to a combination of isoflurane (0.5%) and continuous IV drip of Ketacine® (Ketamine HCl, 0.25 mg/(kg*min), Putney, Boise, ID, USA) in all pigs throughout the entire study period. After a 10-min stabilization period, blood samples were taken from the femoral artery and the right jugular vein for baseline measurements. The urine bag was emptied before urine output measurement started at baseline. Hemorrhage was then induced by bleeding from the femoral artery into sterile empty blood bags containing standard anticoagulant citrate phosphate dextrose solution. The pigs were hemorrhaged 60% of their estimated total blood volume exponentially over 60 min using a computer-controlled pump as described in our laboratory [24]. Upon the completion of hemorrhage, pigs were randomly assigned to LR or NS group. In the LR group, pigs were resuscitated with LR at 3 times the bled volume given over a 45-min period. In the NS group, resuscitation with NS was given to reach the same MAP as achieved after resuscitation in the LR group. Therefore, an NS resuscitated pig was done after a LR resuscitated pig. The LR of 3 times the bled volume was used since this ratio was conventionally recommended as initial fluid resuscitation of hemorrhagic shock for prehospital trauma life support [25]. Pigs in the sham control group were not hemorrhaged or resuscitated. Pigs in all three groups were given the same amount of anesthesia and maintenance fluid (0.04 ml/kg/min) and no significant changes in hemodynamics and coagulation were observed in the sham control pigs during the entire study period, suggesting the stability of the anesthesia regimen. The entire study was depicted in Figure 1. No heparin was used in this study.

All pigs were observed for 6 h after the completion of resuscitation. Blood samples were taken at baseline, after hemorrhage, 15 min, 3 h, and 6 h after resuscitation for measurements of blood gas, blood chemistry, and coagulation (Figure 1). At the end of 6 h, surviving animals were euthanized with Fatal-Plus® (sodium pentobarbital, 100 mg/kg, Vortech, Dearborn, MI, USA, intravenously).

### Hemodynamic and metabolic measures

MAP and heart rate were recorded continuously during the study. Cardiac output was determined by thermodilution in triplicate at baseline, at the end of hemorrhage, and then hourly throughout the remainder of the study. Stroke volume (SV) was calculated as cardiac output (ml/min)/heart rate (beat/min). Total peripheral resistance was calculated as (MAP-CVP)(mmHg)/CO (L/min).

Oxygen delivery was calculated as cardiac output (L/min) × arterial oxygen content (ml O_2_/100 ml blood) × 10/body wt (kg). Oxygen consumption (VO_2_) was calculated from the arterial (C_a_O_2_) and mixed venous (C_v_O_2_) oxygen content using established formulas. Percentage oxygen extraction was calculated as (C_a_O_2_ – C_v_O_2_)/C_a_O_2_. As we described previously [26], oxygen demand was calculated as plasma lactate (mmol/L from the arterial blood gas sample) + oxygen consumption (ml O_2_/min/kg), using the assumption as per Hannon and colleagues [27]. The oxygen delivery to oxygen demand ratio, an index of oxygen debt, was calculated as oxygen delivery/oxygen demand.

### Analytical methods

Measurements of blood gas [pH, base excess, lactate, hematocrit (Hct), bicarbonate (HCO_3_^-^), etc.] and blood chemistry (total protein, electrolytes, etc.) were determined by standard clinical laboratory analysis. Hct and platelet counts were measured from citrated blood using an ABX Pentra 120 Hematology Analyzer (ABX Diagnostics, Inc., Irvine, CA). Plasma fibrinogen concentration, prothrombin time (PT), and activated partial prothrombin time (aPTT) were measured using the BCS Coagulation System (Dade Behring, Deerfield, IL).

The coagulation profile was assessed in fresh whole blood with pig thromboplastin by thrombelastograph® (TEG) using a TEG 5000 Hemostasis Analyzer (Haemoscope, Niles, IL) In TEG measurements, reaction time (R time) is the latency time for initial clot formation, K time reflects the speed to reach a certain level of clot strength, angle α reflects the rapidity of clot build-up and cross-linking, MA represents the maximum strength or stiffness of the clot, and LY_60_ indicates the percentage of clot lysis at 60 min after maximum clotting is achieved.

### Statistical analysis

Data were expressed as means ± standard error of the mean (SEM) and analyzed using SAS statistical software (Cary, NC). A two-way analysis of variance (ANOVA) with repeated measures using a Tukey adjustment was used to compare the changes over time between the groups. A one-way ANOVA with repeated measures using a Dunnett adjustment was used to compare the changes to baseline within the group. The statistically significant level was set at p < 0.05.

## Results

### Hemodynamics, cardiac-pulmonary function, and oxygen metabolic responses

**Figure 2:**
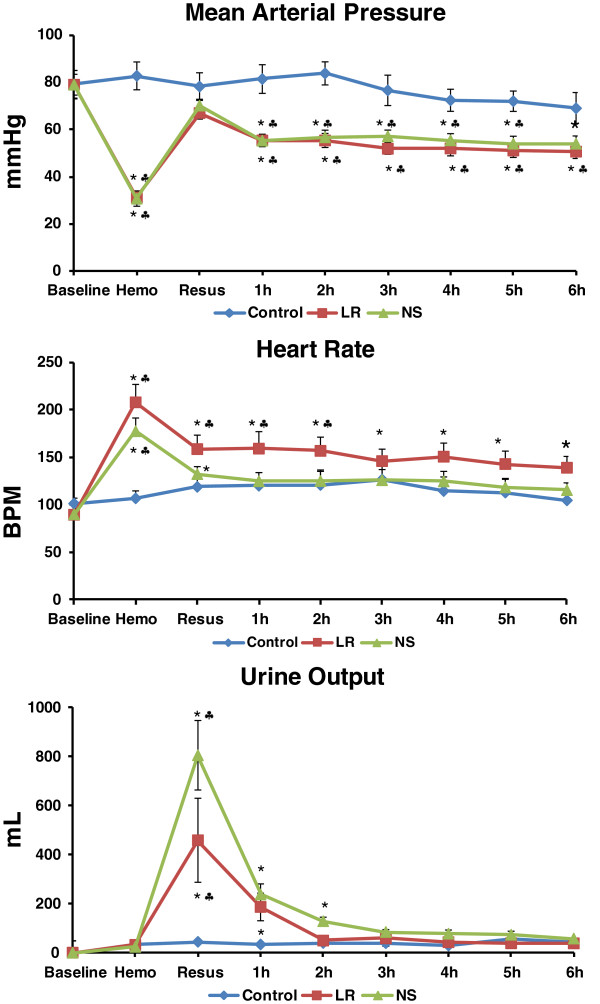
**Changes in mean arterial pressure (MAP), heart rate (HR), and urine output after hemorrhage and resuscitation with lactated Ringer’s solution (LR, 119 ± 7 ml/kg) or normal saline (NS, 183 ± 9 ml/kg) in pigs.** *p < 0.05 compared to the corresponding baseline values. ^♣^p < 0.05 compared to corresponding control values.

All animals survived to the end of the 6 h study. No significant changes in the hemodynamics were observed in the control group over the experimental period. A 60% hemorrhage dropped MAP from the baseline value of 79 ± 6 mm Hg to 31 ± 3 mm Hg (p < 0.05). In the LR group, resuscitation with LR at 3 times bled volume (119 ± 7 ml/kg) led to a MAP that peaked briefly near pre-hemorrhage levels, but then fell and remained at hypotensive levels around 60 mmHg (Figure 2). In the NS group, 183 ± 9 ml/kg of NS was infused to achieve MAP similar to that in the LR group (Figure 2). Heart rate was increased by hemorrhage, followed by a partial decline after resuscitation in both groups. Heart rate returned to near baseline levels in the NS group, but remained higher over baseline in the LR group (Figure 2). Urine output rose with resuscitation in both groups with a larger increase in the NS group, followed by a decline to baseline values at 2 h after resuscitation in the LR group (Figure 2). From the start of resuscitation to 15 min after the completion of resuscitation, urine output in the NS group (23 ± 5 ml/kg) doubled that in the LR group (10 ± 3 ml/kg, with 1 ± 0 ml/kg in the control group). The total urine output over the 6 h resuscitation period in the NS group (45 ± 5 ml/kg) also doubled that in the LR group (21 ± 4 ml/kg, with 10 ± 1 ml/kg in the control group, all p < 0.05 LR versus NS, and LR or NS versus control).

No significant changes in cardiac output, stroke volume, total peripheral resistance, oxygen content, or oxygen extraction were observed in controls during the experiment (Table 1). No significant changes in temperature were observed in any groups during the study. Cardiac output returned to pre-hemorrhage levels at the end of LR resuscitation (15 min). In contrast, cardiac output was increased 50% over BL by NS resuscitation and remained elevated in this group for the 6 h experimental period. Stroke volume returned to pre-hemorrhage levels with NS resuscitation (15 min), but remained below pre-hemorrhage levels for the 6 h experimental period after LR resuscitation (Table 1). Total peripheral resistance was decreased after resuscitation with LR or NS, with a lower resistance shown in the NS group vs. the LR group (Table 1).

**Table 1:** Changes of cardiac-pulmonary functions and hemodilution after hemorrhage and resuscitation with lactated Ringer’s (LR) solution or normal saline (NS) in pigs

	**Baseline**	**Resuscitation**	**3 h**	**6 h**
**Cardiac output** (L/min)				
Control	3.9 ± 0.3	4.3 ± 0.3	4.4 ± 0.4	3.6 ± 0.2
LR	3.7 ± 0.2	3.9 ± 0.3	3.5 ± 0.2	3.3 ± 0.2
NS	3.8 ± 0.3	5.9 ± 0.5*^§♣^	4.7 ± 0.3^§^	4.5 ± 0.2^§♣^
**Stroke volume** (ml/beat)				
Control	36.5 ± 3.5	36.8 ± 2.2	36.8 ± 3.7	35.5 ± 3.9
LR	41.4 ± 3.3	24.9 ± 1.6*^§♣^	21.4 ± 2.3*^§^	24.7 ± 2.1*^§^
NS	38.2 ± 4.9	45.7 ± 4.7	38.1 ± 3.3	40.1 ± 3.3
**Total Peripheral resistance** (mm Hg/(L/min))				
Control	20.4 ± 1.4	18.6 ± 2.0	17.3 ± 2.6	17.7 ± 1.3
LR	22.9 ± 1.0	17.7 ± 0.9*	17.9 ± 1.3*	17.4 ± 0.7*
NS	23.1 ± 1.8	12.5 ± 1.4*^§^	12.5 ± 0.7*^§^	12.0 ± 0.4*^§^
**Hct (%)**				
Control	29.0 ± 0.8%	28.9 ± 0.9%	28.0 ± 0.9%	27.6 ± 1.0%
LR	30.2 ± 1.0%	13.1 ± 0.9%*^♣^	14.0 ± 0.7%*^♣^	14.7 ± 0.6%*^♣^
NS	29.0 ± 1.0%	11.6 ± 0.8 %*^♣^	12.5 ± 0.6%*^♣^	12.2 ± 0.7%*^♣^

Animal groups include control (C, n = 6), hemorrhage with LR resuscitation (LR, n = 6), and hemorrhage with normal saline resuscitation (NS, n = 6). Data are expressed as means ± SE.

*p < 0.05 compared to corresponding baseline values within the group; ^§^p < 0.05 LR vs NS groups; and ^♣^p < 0.05 compared to corresponding control values.

**Figure 3:**
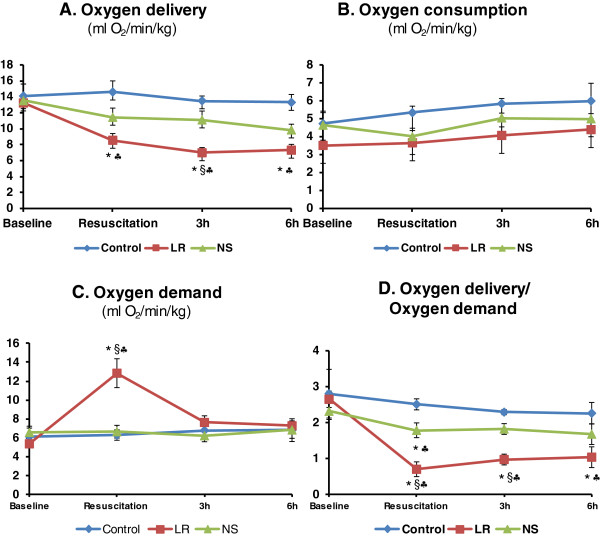
**Changes in oxygen delivery (A), oxygen consumption (B), oxygen demand (C), and oxygen delivery/oxygen demand (D) after hemorrhage and resuscitation with lactated Ringer’s solution (LR, 119 ± 7 ml/kg) or normal saline (NS, 183 ± 9 ml/kg) in pigs.** *p < 0.05 compared to the corresponding baseline values. ^§^p < 0.05 LR group compared to NS group. ^♣^p < 0.05 compared to corresponding control values.

Oxygen metabolic responses to hemorrhage and resuscitation with LR or NS were described in Table 2 and Figure 3. Arterial PO_2_ and PCO_2_ were similar among the 3 groups during the study (Table 2). Venous PO_2_ returned to pre-hemorrhage levels 15 min after LR or NS resuscitation, but declined during the remaining 6 h with a larger decrease observed in LR group (Table 2). There were no differences in venous PCO_2_ among the 3 groups during the study (Table 2). Arterial oxygen content was decreased for 6 h after hemorrhage and resuscitation with LR or NS (Table 2). Venous oxygen content was also decreased for 6 h after hemorrhage and resuscitation with LR or NS (Table 2). Oxygen extraction was increased for 6 h after resuscitation with LR solution or NS. Oxygen delivery was reduced after resuscitation with LR, with no significant changes observed in the NS group (Figure 3A). Oxygen consumption remained unchanged in all three groups during the study (Figure 3B). Oxygen demand did not change after NS resuscitation, but doubled immediately after LR resuscitation, but returned to baseline values by 3 h (Figure 3C). Oxygen delivery to oxygen demand ratio, an index of oxygen debt, was decreased after NS resuscitation but returned to baseline value within 3 h, whereas a larger drop was observed after LR resuscitation and remained low over the course of the study (Figure 3D).

**Table 2:** Changes in oxygen content and extraction after hemorrhage and resuscitation with lactated Ringer’s (LR) solution or normal saline (NS) in pigs

	**Baseline**	**Resuscitation**	**3 h**	**6 h**
**Arterial O**_**2**_ (mmHg)				
Control	475 ± 18	460 ± 15	497 ± 16	470 ± 15
LR	493 ± 15	513 ± 10	515 ± 15	499 ± 11
NS	478 ± 25	474 ± 21	490 ± 17	470 ± 16
**Arterial CO**_**2**_ (mmHg)				
Control	47 ± 4	48 ± 4	45 ± 3	45 ± 4
LR	44 ± 3	43 ± 3	45 ± 3	43 ± 2
NS	47 ± 2	43 ± 2	44 ± 2	44 ± 1
**Venous O2** (mmHg)				
Control	55 ± 5	60 ± 4	58 ± 5	50 ± 5
LR	56 ± 2	53 ± 3	36 ± 1*^♣^	36 ± 1*^♣^
NS	54 ± 2	53 ± 2	46 ± 2*^♣^	43 ± 1*^♣§^
**Venous CO2** (mmHg)				
Control	60 ± 5	57 ± 4	50 ± 3	51 ± 5
LR	53 ± 2	50 ± 2	56 ± 2	56 ± 1
NS	59 ± 5	50 ± 2	55 ± 1	55 ± 1
**Arterial oxygen content** (ml O_2_/dL blood)				
Control	13.8 ± 0.6	13.8 ± 0.5	13.6 ± 0.3	13.3 ± 0.7
LR	14.7 ± 0.3	7.6 ± 0.3*^♣^	8.5 ± 0.6*^♣^	7.8 ± 0.4*^♣^
NS	13.2 ± 0.9	6.4 ± 0.4*^♣^	7.9 ± 0.4*^♣^	7.3 ± 0.4*^♣^
**Venous oxygen content** (ml O_2_/dL blood)				
Control	9.9 ± 0.5	9.9 ± 0.7	9.3 ± 0.6	7.7 ± 0.9
LR	10.8 ± 0.2	4.3 ± 0.2*^♣^	3.5 ± 0.2*^♣^	3.1 ± 0.2*^♣^
NS	8.6 ± 0.6	4.1 ± 0.3*^♣^	4.2 ± 0.3*^♣^	3.8 ± 0.3*^♣^
**Oxygen extraction** (%)				
Control	28 ± 4	29 ± 3	32 ± 3	32 ± 3
LR	28 ± 2	43 ± 2*	57 ± 3*	60 ± 3*
NS	35 ± 5	36 ± 3	47 ± 3*	49 ± 3*

Animal groups include control (C, n = 6), hemorrhage with LR resuscitation (LR, n = 6), and hemorrhage with normal saline resuscitation (NS, n = 6). Data are expressed as means ± SE.

*p < 0.05 compared to corresponding baseline values within the group; ^§^p < 0.05 LR vs NS groups; and ^♣^p < 0.05 compared to corresponding control values.

### Hemodilution, acid base balance, and electrolytes

No significant changes in Hct, base excess, or lactate concentrations were observed in the control group over the entire experimental period. Significant hemodilution was observed after hemorrhage and resuscitation with LR or NS (Table 1). Total protein dropped from baseline value of 5.6 ± 0.2 g/dL to 2.7 ± 0.1 g/dL at 15 min and 3.3 ± 0.1 g/dL at 6 h after LR resuscitation and from baseline value of 5.7 ± 0.2 g/dL to 3.0 ± 0.2 g/dL at 15 min and 3.5 ± 0.2 g/dL at 6 h after NS resuscitation (all p < 0.05 vs. baseline). There were no significant differences in Hct (Table 1) or total protein between LR and NS groups during the 6 h after resuscitation.

**Figure 4:**
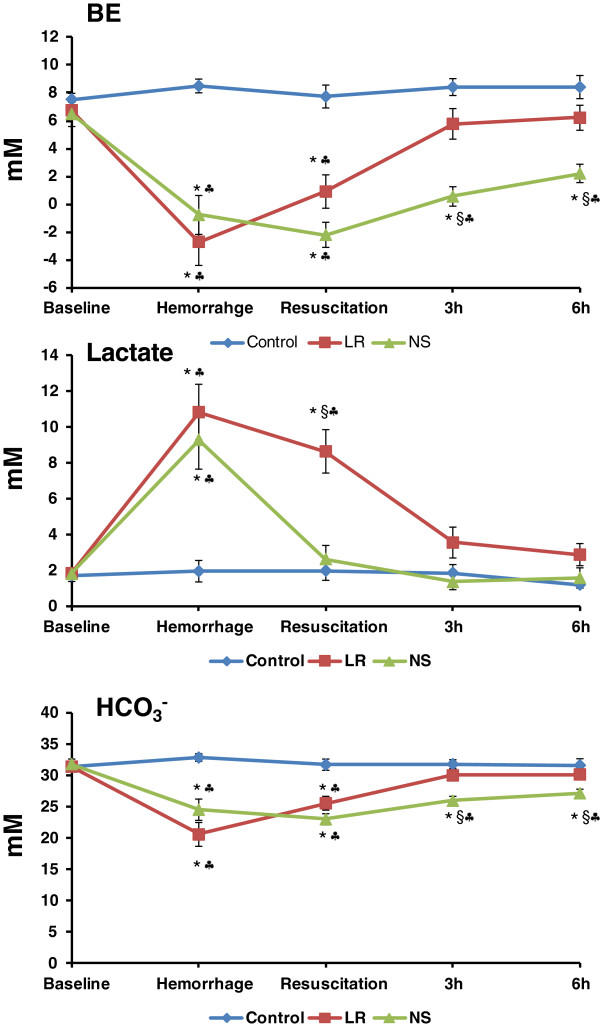
**Changes in acid-base status after hemorrhage and resuscitation with lactated Ringer’s solution (LR, 119 ± 7 ml/kg) or normal saline (NS, 183 ± 9 ml/kg) in pigs.** *p < 0.05 compared to the corresponding baseline values. ^§^p < 0.05 LR group compared to NS group. ^♣^p < 0.05 compared to corresponding control values.

Base excess fell below zero after hemorrhage and returned to pre-hemorrhage levels with LR resuscitation by 3 h but remained below pre-hemorrhage levels for 6 h with NS resuscitation (Figure 4). Plasma lactate levels rose about 4.5-fold above baseline values by hemorrhage and returned to pre-hemorrhage value within 15 min after NS resuscitation and at 6 h after LR resuscitation (Figure 4). Bicarbonate HCO_3_^-^ levels were decreased by hemorrhage but returned to pre-hemorrhage values by 3 h after LR resuscitation, whereas no return was observed with NS resuscitation (Figure 4). Arterial pH was decreased from a pre-hemorrhage value of 7.41 ± 0.02 to 7.35 ± 0.01 after NS resuscitation (p < 0.05) but returned to pre-hemorrhage value at the 3 h time point. No significant changes in arterial pH occurred in the control or LR group during the study.

There were no significant changes in Na^+^, K^+^, Ca^++^, or Cl^-^ in the control group during the entire study period (Table 3). Na^+^ was increased after NS resuscitation but returned to pre-hemorrahge level within 6 h. K^+^ did not change initially after NS resuscitation but was elevated at 6 h afterwards (Table 3). No changes in Na^+^ or K^+^ were observed in pigs with LR resuscitation (Table 3). Ca^++^ was similarly decreased at 15 min after resuscitation with LR or NS but returned to pre-hemorrhage levels by 6 h in both groups (Table 3). Cl^-^ was elevated for 6 h after NS resuscitation, with no changes shown after LR resuscitation (Table 3).

**Table 3:** Changes of electrolytes after hemorrhage and resuscitation with lactated Ringer’s (LR) solution or normal saline (NS) in pigs

	**Baseline**	**After Hemorrhage and Resuscitation**	**6 h**
**Na**^**+**^ (mM)			
Control	138 ± 3	139 ± 6	141 ± 2
LR	140 ± 3	138 ± 3	132 ± 3
NS	141 ± 2	150 ± 3^*§♣^	144 ± 6
**K**^**+**^ (mM)			
Control	4.5 ± 0.2	4.7 ± 0.4	4.6 ± 0.2
LR	4.3 ± 0.2	4.3 ± 0.2	5.2 ± 0.4
NS	4.5 ± 0.2	4.5 ± 0.3	5.7 ± 0.3*
**Ca**^**++**^ (mM)			
Control	9.2 ± 0.2	9.4 ± 0.3	8.9 ± 0.1
LR	9.5 ±0.4	7.7 ± 0.3^*♣^	8.3 ± 0.8
NS	9.4 ± 0.3	7.9 ± 0.2^*♣^	9.0 ± 0.3
**Cl **^**-**^ (mM)			
Control	97 ± 2	109 ± 8	100 ± 1
LR	101 ± 2	102 ± 2	97 ± 3
NS	102 ± 3	121 ± 6*	123 ± 6*

Animal groups include control (C, n = 6), hemorrhage with LR resuscitation (LR, n = 6), and hemorrhage with normal saline resuscitation (NS, n = 6). Data are expressed as means ± SE.

*p < 0.05 compared to corresponding baseline values within the group; ^§^p < 0.05 LR vs NS groups; and ^♣^p < 0.05 compared to corresponding control values.

### *Coagulatio*n

Plasma fibrinogen concentration in control pigs remained unchanged during the study. Plasma fibrinogen concentration was decreased from a baseline value of 193 ± 12 mg/dL to 95 ± 5 mg/dL after LR resuscitation (p < 0.05) and from a baseline value of 189 ± 9 mg/dL to 101 ± 5 mg/dL after NS resuscitation (p < 0.05). Platelet count was decreased from the baseline value of 359 ± 54 10^3^/μL to 172 ± 28 10^3^/μL after LR resuscitation and from a baseline value of 427 ± 58 10^3^/μL to 207 ± 29 10^3^/μL after NS resuscitation (both p < 0.05). There were no significant differences between LR and NS groups in fibrinogen concentrations or platelet count during the 6 h after resuscitation.

**Figure 5:**
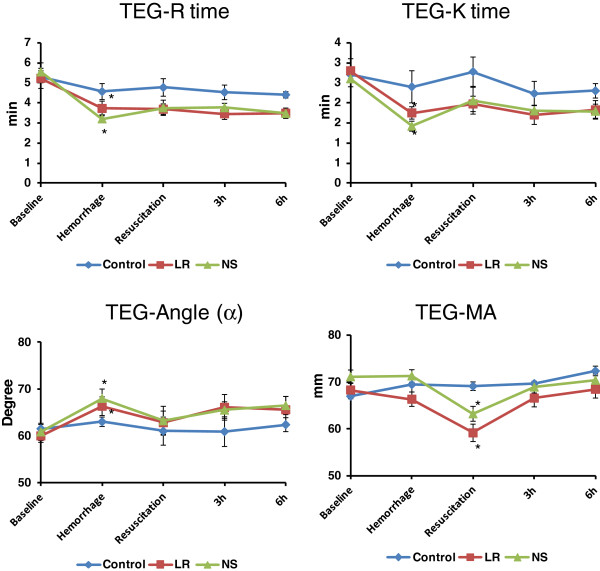
**Changes in Thrombelastograph® (TEG) measurements after hemorrhage and resuscitation with lactated Ringer’s solution (LR, 119 ± 7 ml/kg) or normal saline (NS, 183 ± 9 ml/kg) in pigs.** R time: latency time for initial fibrin formation. K time: a measure of speed to reach a certain level of clot strength. Angle a: the rapidity of fibrin build up and cross-linking. MA: maximum strength of the developed clot. *p < 0.05 compared to the corresponding baseline values.

There were no changes in TEG measurements in the control group during the study (Figure 5). R time and K times were shortened by hemorrhage but returned to near pre-hemorrhage values after resuscitation with LR or NS (Figure 5). Clotting speed (α-angle) was increased by hemorrhage and returned to pre-hemorrhage values after LR or NS resuscitation (Figure 5). Clot strength (MA) was not changed by hemorrhage but was similarly reduced by resuscitation with LR or NS, followed by return to baseline values within 3 h after resuscitation (Figure 5). No significant changes in fibrinolysis (LY_60_) were observed in any group during the study (data not shown).

Plasma PT or PTT was not changed by hemorrhage. PT was similarly prolonged by resuscitation with LR (from 11.2 ± 0.2 sec at baseline to 12.1 ± 0.2 sec at 6 h) and NS (from 11.2 ± 0.3 sec at baseline to 12.3 ± 0.3 sec at 6 h, both p < 0.05). Plasma aPTT was also similarly prolonged by resuscitation with LR (from 17.1 ± 0.5 sec baseline to 20.1 ± 1.2 sec at 6 h) or NS (from 17.1 ± 0.3 sec baseline to 19.1 ± 0.5 sec at 6 h, both p < 0.05).

## Discussion

Using a large-animal model with severe hemorrhage and 6 h follow-up, we performed a comprehensive assessment of the physiologic and biochemical effects of LR and NS. To achieve a similar hypotensive resuscitation level 50% more volume of normal saline was required than LR to achieve similar responses in MAP. Hemodilution, however, was not different between groups indicating that that the additional NS did not stay in the vascular, as supported by the greater urine output in the NS group. In addition, NS resulted in a lower peripheral resistance and a higher stroke volume, possibly due to its vasodilation effects. Despite similar oxygen extraction and oxygen consumption, NS resuscitation resulted in better oxygen delivery and oxygen delivery-to-oxygen demand ratio as an index of oxygen debt. This better response appears to be primarily due to vasodilation effects as suggested by the large increase in cardiac output compared to the LR group. Thus, in the current severe hemorrhage model, NS had better tissue perfusion and oxygen metabolism than LR. However, since the oxygen delivery to demand ratio was 1 or higher in the LR and NS groups at 6 hrs, both fluids seemed adequate in maintaining survival at least through 6 h.

Significant separation was observed between LR and NS in acid base balance in this study, as expected. LR resuscitation returned BE and bicarbonate to pre-hemorrhage levels within 3 h, but no return of BE or bicarbonate was observed for 6 hr with NS resuscitation. Similar changes of BE and bicarbonate from LR or NS have been shown in different animal models and in patients [18,19,28]. The impact of acid base status from resuscitation fluids on survival has been assessed by Traverso et al. in an anaesthetized swine model with fatal hemorrhage [18]. With equal volume resuscitation from NS (pH 5.0), LR (pH 6.5, 28 mM lactate), and Plasmalyte-A (pH 7.4, 27 mM acetate), the LR group had the best survival and the Plasmalyte-A had the worst survival rate [18]. But BE and pH from LR and Plasmalyte-A resuscitation were similar and higher than those from NS [18], suggesting a better acid base status was not necessarily attributable to a better survival. Nevertheless, as an index of shock, improved BE should be a goal to improve outcome and acidosis is associated with well-known detrimental effects on the cardiovascular system and coagulation [29,30].

Clinical trials have failed to show significant differences in outcomes of LR and NS resuscitation. In comparative trials of LR and NS performed in patients who underwent abdominal aortic aneurysm repair or renal transplant, Waters et al. [15] and Khajavi et al. [28] reported that there were no differences in clinical outcomes, such as duration of ventilation, ICU stay, or incidence of complication between patients resuscitated with LR or NS, although the patient group with NS resuscitation was more acidotic. Using a rat model with hemorrhage and simultaneous resuscitation, Healey et al. found that NS and LR had equivalent survival rates under moderate hemorrhage (36% of estimated blood volume). But with a massive hemorrhage (218% of estimated blood volume), LR resuscitation resulted in better survival [17]. Thus, it appears that the separation of outcomes from LR and NS becomes apparent only under extreme circumstance, such as when greater than 1 blood volume of fluid is given.

Lactate rose about 5 fold after 60% hemorrhage in this study and returned to pre-hemorrhage levels after resuscitation with LR or NS. The recovery rates, however, were different: NS returned lactate to pre-hemorrhage level within 15 min, but lactate did not return to near pre-hemorrhage level for at least 3 h after resuscitation. At 15 min after resuscitation, lactate levels were 8.7 ± 1.2 mM in the LR group and 2.6 ± 0.8 mM in the NS group. This difference could result from lactate load from LR resuscitation or from a larger-volume dilution from NS resuscitation. Calculation of fluid inflow and outflow suggested that the latter is unlikely. From the start of resuscitation to 15 min after the completion of resuscitation, a total of 4.6 ± 0.3 L of LR was infused and urine output was 0.46 ± 0.15 L in the LR group, with a net fluid inflow of 4.1 L. During the same period a total of 6.1 ± 0.1 L of NS was infused for resuscitation while urine output was 0.81 ± 0.18 L, with a net fluid inflow of 4.9 L. This 20% volume difference (4.1 L of LR vs. 4.9 L of NS) would not be able to fully explain the observed 3.5fold difference of lactate between the LR (8.7 mM) and NS (2.6 mM) group. Thus, the difference of lactate levels at 15 min after resuscitation may relate to the lactate load from LR resuscitation, at least in part. However, this difference gradually disappeared within 6 h with a simultaneous increase of HCO_3_^-^ in the LR group, suggesting that an ongoing lactate metabolism in pigs and the production of bicarbonate may contribute to the better acid-base status from lactate resuscitation. Since the metabolism of lactate in humans takes a few hours and involves producing H^+^ and HCO_3_^-^[31], a similar time frame observed in the present study indicates similar lactate metabolism in humans and pigs. In addition, current blood bank guidelines state that LR should not be mixed with blood to prevent the risk of clot formation from calcium included in LR, which can diminish the anti-coagulation effect in stored blood. Thus, LR resuscitation should not be given with blood through the same iv-line and crystalloids should be avoided in patients with blood transfusion.

Hemorrhage and resuscitation caused disturbances in the coagulation process. PT and aPTT were prolonged for 6 h after hemorrhage and resuscitation, suggesting a hypocoagulable states. In contrast, the TEG data suggested a hypercoagulable states. These differences between TEG and standard plasma assays were also observed in burn and trauma patients [32,33]. Clotting initiation was shortened, and clotting speed was accelerated by hemorrhage; but both returned to pre-hemorrhage values after resuscitation. Clot strength was compromised after resuscitation but returned within 3 h. Despite these dynamic changes in the coagulation profile, there were no differences between LR and NS resuscitation during the study, suggesting the equivalent effects of the two fluids on coagulation. In contrast, a faster clotting speed and better clot strength from LR resuscitation were reported by Kiraly et al. [19]. This discrepancy is likely due to the differences in study design. The present study used a swine model with controlled hemorrhage with 50% more volume of NS, whereas Kiraly et al. used a swine model of liver injury and an uncontrolled hemorrhage with 150% more volume of NS used. The additional hemodilution from the 150% NS might contribute to the different findings. In addition, a sham control group was included in the present study and our data showed that coagulation profiles from LR and NS resuscitation returned to pre-hemorrhage values and became similar to those of the control values. Thus, potential thrombotic risk from LR resuscitation is unlikely.

In order to make a valid comparison, a common physiological endpoint after resuscitation is needed to assess the effects of resuscitation. In this study, we used post-resuscitation MAP as the physiological endpoint to compare the effects of LR and NS on hemodynamics, coagulation and metabolism. We found that NS required 50% more volume to reach the same physiological endpoint. If the same volume of resuscitation was used in LR and NS groups, we suspected that the blood pressure after NS resuscitation would be lower than that of LR due to its vasodilator effects.

A fixed volume controlled hemorrhage model was used in this study to investigate metabolic, hemodynamic and coagulation effects of hypovolemia and fluid resuscitation. This model mimics situations where trauma patients bleed an amount of blood before hemorrhage is controlled [34]. However, it is limited in reflecting coagulation changes under a clinical scenario of continued bleeding (or rebleeding). It is likely the on-going bleeding from uncontrolled hemorrhage will exaggerate the impact of vasodilation effects from NS, with higher volumes infused and more hemodilution and coagulation impairment. Considering that restriction on resuscitation fluid has been shown improving survival in uncontrolled bleeding patients [35] and animals [36], a larger volume of NS, as compared to LR, would increase bleeding with resultant higher morbidities and mortality in trauma patients.

In this study, to reach the same resuscitative physiological endpoint, NS required 50% more volume and was associated with a higher cardiac output and lower peripheral resistance, as compared to LR resuscitation. These differences are possibly due to the vasodilator effects from NS. It is worth emphasizing that these effects are likely to be exaggerated under uncontrolled hemorrhage, resulting in more fluid volume, a larger hemodilution, and more severe impairment in coagulation. Further, an elevation of K^+^ was observed at 6 h post NS resuscitation, while no change of K^+^ was observed after LR resuscitation. The mechanism for the increase of K^+^ from NS is not fully known, but may be due to an extracellular shift of potassium caused by changes in blood hydrogen ion concentration, as the result of hyperchloremic acidosis from NS resuscitation [37]. Consistent with our current results, clinically significant hyperkalemia from NS administration has been reported in patients during renal transplantation [16,28]. Thus, NS is associated with vasodilator effects and the risks of metabolic acidosis and hyperkalemia. Currently, military first responders have NS, LR and Hextend available [20]. However, the results from the current study and our recent study [38] suggest that all of these fluids have limitations in managing hemodynamics, metabolic responses, and coagulation. The optimum resuscitation fluid awaits further development.

## Conclusions

We compared resuscitative effects of NS and LR in a swine model with 60% controlled hemorrhage. Although LR and NS had equivalent effects on hemodynamics and oxygen metabolism, NS required a larger resuscitation volume and was associated with poor acid base status and elevated serum potassium in this model. Thus, NS may be inferior to LR in resuscitation due to its vasodilator effects and the risks of metabolic acidosis and hyperkalemia.

## Abbreviations

BE: Base excess; BL: Baseline; IV: Intravenous; LR: Lactated Ringer’s; MAP: Mean arterial pressure; NS: Normal saline; PT: Prothrombin time; aPTT: Activated partial prothrombin time; TEG: Thrombelastography^®^; TEG measurements: ; R time: Latency time for initial fibrin formation; K time: a measure of speed to reach a certain level of clot strength; Angle α: the rapidity of fibrin build up and cross-linking; MA: Maximum strength of the clot; LY60: Percentage of clot lysis at 60 min after MA is achieved.

## Competing interests

The authors declare that they have no competing interests.

## Authors’ contributions

WM designed and performed the study, analyzed the data with assistance from statistician Mr John Jones, and wrote the manuscript. DC performed the study. MD designed study and edited the manuscript. All authors read and approved the final manuscript.
